# Bridging algorithmic prediction and clinical agency: an exploratory pilot study of AI-augmented physician antidepressant choice

**DOI:** 10.3389/fpsyt.2026.1851899

**Published:** 2026-07-03

**Authors:** Akiva Kleinerman, David Benrimoh, Amit Yaniv-Rosenfeld, Grace Golden, Myriam Tanguay-Sela, Howard C. Margolese, Teddy Lazebnik, Ben H. Amit, Hadar Samuel, Ariel Rosenfeld

**Affiliations:** 1 Department of Information Science and Applied AI, Bar-Ilan University, Ramat Gan, Israel; 2 Aifred Health, Montreal, QC, Canada; 3 Department of Psychiatry, Stanford University, Stanford, CA, United States; 4 Shalvata Mental Healthcare Center, Hod-HaSharon, Israel; 5 Psychiatry Department, Faculty of Medicine, Tel Aviv University, Tel Aviv, Israel; 6 Department of Computing, Jonkoping University, Jonkoping, Sweden; 7 Department of Information Systems, University of Haifa, Haifa, Israel; 8 Department of Psychology, University of Western Ontario, London, ON, Canada; 9 Department of Psychology, Université du Québec á Montréal, Montreal, QC, Canada; 10 Department of Psychiatry, McGill University, Montreal, QC, Canada; 11 Myers-JDC-Brookdale Institute, Jerusalem, Israel

**Keywords:** artificial intelligence, clinical agency, clinical decision support systems, major depressive disorder, user study

## Abstract

**Introduction:**

Effective psychiatric decision-making requires balancing data-driven predictions with clinical agency. This challenge is particularly acute in the pharmacological management of Major Depressive Disorder (MDD), where clinicians must navigate complex, patient-specific trade-offs between remission probabilities and diverse side-effect risks. Although AI-driven Clinical Decision Support Systems (AI-CDSS) can support the prediction of individual treatment outcomes, the optimal mechanism for aggregating multiple clinical criteria remains an open research challenge.

**Methods:**

We investigated how the locus of control in the aggregation mechanism affects clinical utility and treatment decisions. Three weighting schemes were evaluated: (1) an Implicit Weighting baseline, in which raw probabilities were presented; (2) a Static Expert-Derived Weighting scheme, using linear aggregation with fixed expert-based weights; and (3) a Dynamic Clinician-Determined Weighting scheme, using linear aggregation with adjustable clinician-defined weights. These schemes were implemented within a prototype decision support system for antidepressant selection and evaluated in a user study with 22 physicians.

**Results:**

The Dynamic Clinician-Determined Weighting scheme significantly enhanced perceived clinical utility compared with the alternative approaches (p < 0.01). It also led to the most frequent data-informed revision of physicians’ initial unassisted antidepressant choices, occurring in 33.3% of cases. This effect was observed among both psychiatrists and primary care physicians, suggesting that adjustable weighting can support more informed treatment decisions across clinical specialties.

**Discussion:**

These findings suggest that effective integration of AI into psychiatric practice requires flexible decision support systems that preserve clinical agency while incorporating data-driven predictions. By allowing clinicians to determine the relative importance of remission probabilities and side-effect risks, dynamic weighting may better reflect the nuanced and individualized nature of mental health care.

## 1 Introduction

Clinical Decision Support Systems (CDSS) have evolved from rule-based engines to sophisticated platforms leveraging Artificial Intelligence (AI) to empower data-driven clinical decisions (1, 2). This evolution is particularly critical for mitigating the burden associated with Major Depressive Disorder (MDD), a condition characterized by high prevalence and significant morbidity (3, 4). Despite the availability of pharmacological interventions, the current standard of care often relies on an “educated trial-and-error” approach (5), where only a third of patients achieve remission after their first treatment (6, 7). In this context, AI models have demonstrated significant potential in predicting individual treatment outcomes, including probabilities of remission (5, 8, 9), while pharmacological literature highlights specific adverse events such as weight gain, sexual dysfunction, and fatigue (10–12).

Nevertheless, despite improvements in predictive accuracy, the translation of these models into clinical practice remains limited (13, 14). A fundamental barrier to adoption is that psychiatric prescribing is rarely a straightforward, single-objective task. Instead, it involves delicate clinical judgments regarding potentially conflicting outcomes (12, 15, 16). In antidepressant selection, this challenge is particularly acute as clinicians must delicately balance efficacy against the risk of side effects, trade-offs that vary significantly depending on the clinical context and patient preferences. While AI can accurately predict the *risk* associated with these outcomes, it cannot inherently determine their *subjective value* within the clinician-patient shared decision-making process (10, 17, 18).

Inevitably, a core challenge in designing AI-CDSS is not merely prediction, but the aggregation of multi-dimensional outputs into actionable recommendations (19–21). Existing decision-support approaches in AI-CDSS typically fall into two extremes: (i) *Implicit Weighting*, also known as “Probabilities Alone (PA)”, where systems present raw probabilities derived from computational models. While transparent, this approach forces clinicians to perform the challenging cognitive integration of these probabilities manually; and (ii) *Static Expert-Derived Weighting*, where systems aggregate model outputs using fixed rules, commonly a weighted sum model using expert-defined linear weights (22, 23). While this approach is designed to reduce cognitive load, it effectively limits the clinician’s ability to adapt the decision logic to patient-specific nuances. This creates an inherent tension between maintaining clinician agency at the cost of high cognitive burden (the PA scheme) or accepting streamlined processing at the cost of rigid, generalized decision logic (the static scheme).

To this end, in this study, we address this tension by proposing and evaluating a middle-ground solution: *Dynamic Clinician-Determined Weighting*. By separating algorithmic risk prediction from the physician’s subjective clinical judgment, this approach allows clinicians to explicitly prioritize the trade-offs relevant to the individual patient and dynamically adjust them as clinical needs evolve (24). We compare this clinician-determined weighting scheme against both implicit and static alternatives through a controlled user study with 22 physicians. Participants interacted with an AI-CDSS prototype across diverse clinical scenarios, allowing us to assess how the locus of control in weighting mechanisms influences the perceived utility and data-informed clinical decisions. The methodological contribution of this exploratory pilot is the direct comparison of three alternative loci of control for aggregating multi-dimensional AI predictions: no explicit aggregation, static expert-defined aggregation, and dynamic clinician-controlled aggregation. This design isolates the role of value-weighting control in AI-CDSS use, a design dimension that is often implicit in decision-support systems but central to preserving clinical agency in preference-sensitive psychiatric decisions.

## 2 Methodology

To study the impact of weighting schemes on clinical decision-making, we conducted a proof-of-concept, within-subjects clinician pilot study (*N* = 22) comparing three distinct weighting schemes implemented to support antidepressant selection. The study was designed to assess how the locus of control (implicit, expert-determined, or clinician-determined) influences clinicians’ perceived utility and treatment selection.

### 2.1 Experimental design and procedure

**Figure 1:**
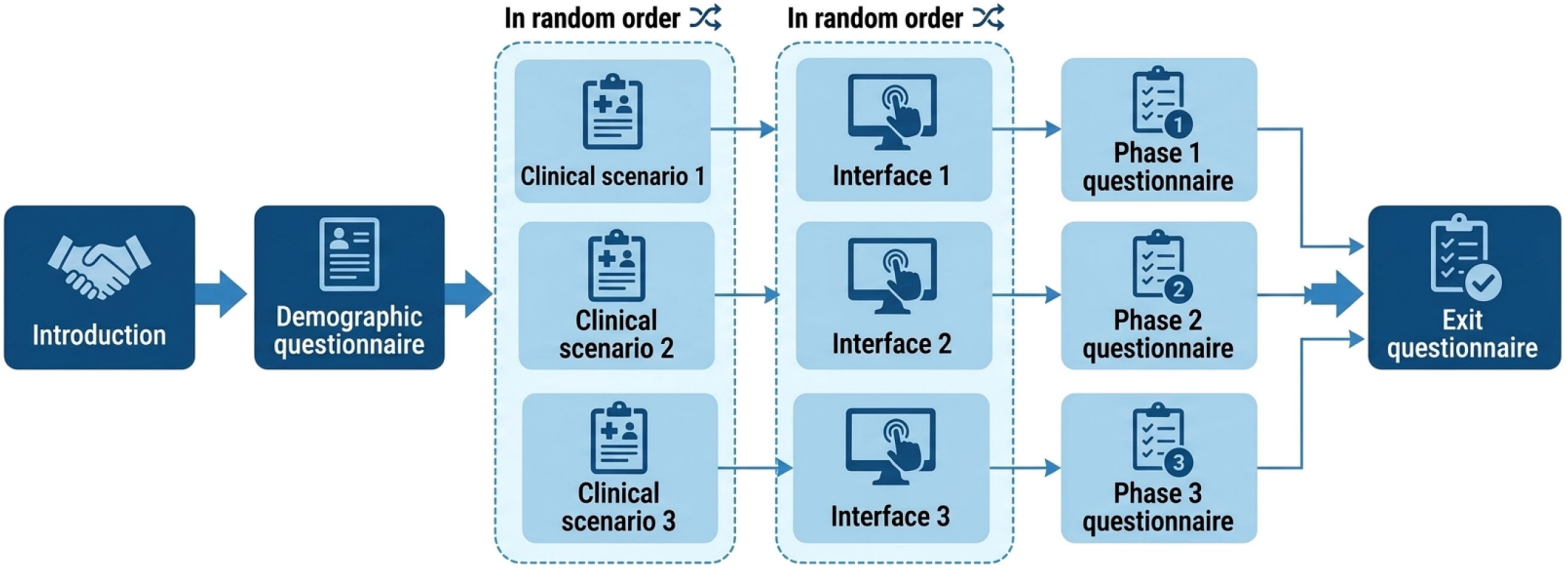
Experimental design. Participants completed three phases in random order, interacting with the three weighting schemes in a randomized sequence. At each phase, participants selected a treatment before and after interacting with the assigned weighting scheme and completed a utility assessment questionnaire. Finally, an exit questionnaire was administered.

The experiment employed a randomized, within-subjects design (25). Data were collected between June and August 2022. The study was conducted online using the web-based prototype; no in-person attendance was required. All participants were offered the option to schedule a video conference with the first author for assistance in case they had any questions. Three participants elected to participate via video conference. Following informed consent and a standard demographic questionnaire, participants completed three distinct decision-making phases. In each phase, participants were presented with: (1) one of the three clinical vignettes described below, and (2) one of the three examined weighting schemes integrated within an AI-CDSS. The pairing of clinical scenarios and weighting schemes was randomized to control for order and case-difficulty effects. **Figure 1** presents a schematic view of the methodological process utilized in this study.

At each phase, participants first reviewed the patient profile. Crucially, *before* interacting with any form of decision support for that patient, they were asked to record their *unassisted* antidepressant choice based on the clinical vignette alone. They then interacted with a prototype AI-CDSS integrated with one of the three weighting schemes to generate recommendations and select a final antidepressant. Following each phase, participants completed a very short questionnaire containing only two questions: “How would you rate the clinical usefulness of the decision support you just experienced? (five point Likert-sclae: 1=not very useful, 5=very useful)” and “Did you change your initial treatment selection following the decision support? (Yes/No)”. A concluding (exit) questionnaire was also administered to probe for comparative preferences and open-form qualitative feedback.

### 2.2 Clinical scenarios

The AI-CDSS was applied to three realistic clinical profiles (“Jack”, “Emma”, and “Sara”) representing mild, moderate, and severe depression, respectively (complete details are provided in the Appendix). Briefly, Jack was an older adult with mild–moderate depression, comorbid hypertension and diabetes, sleep disturbance, fatigue, and concern about weight gain; Emma was a middle-aged professional with moderate–severe depression, work-related stress, passive suicidal ideation without intent or plan, and high concern about sexual dysfunction and weight gain; and Sara was a patient with severe depression, marked functional impairment, psychomotor slowing, frequent suicidal ideation without an active plan, and general concerns about side effects. The probability estimates for each profile (**Table 1**) were constant across all experimental conditions, maintaining the analytical focus on the weighting scheme and its perceived clinical utility and locus of control.

**Table 1:** Predicted probabilities for depression profiles.

Use-case	Medication	Remission	Weight gain	Sexual dysf.	Fatigue
Jack (Mild Depression)	Escitalopram	81%	5%	7%	7%
	Sertraline	76%	6%	20%	10%
	Bupropion	69%	1%	3%	8%
	Venlafaxine	79%	8%	8%	16%
	Mirtazapine	75%	20%	1%	22%
Emma (Moderate Depression)	Escitalopram	42%	3%	17%	6%
	Sertraline	46%	5%	25%	11%
	Bupropion	39%	1.20%	5%	3%
	Venlafaxine	44%	6.50%	10%	8%
	Mirtazapine	41%	50%	3%	35%
Sara (Severe Depression)	Escitalopram	31%	3%	7%	5%
	Sertraline	34%	4%	10%	12%
	Bupropion	24%	1.50%	4%	3%
	Venlafaxine	30%	6%	12%	10%
	Mirtazapine	26%	45%	4%	40%

### 2.3 AI Decision support and evaluation conditions

We developed a web-based AI-CDSS framework aimed at supporting complex decision-making among five common antidepressants (Escitalopram, Sertraline, Bupropion, Venlafaxine, and Mirtazapine). This set of antidepressants represents pharmacological diversity and includes commonly used antidepressants evaluated in large comparative antidepressant evidence syntheses (26, 27). To isolate the impact of the weighting mechanism, the framework was designed to simulate the output of a predictive AI model tailored for MDD treatment. The system presented realistic, pre-defined probability estimates for remission and the risk of three major side effects: weight gain, sexual dysfunction, and fatigue. These estimates, informed by pharmacological literature and expert consensus for three realistic patient profiles (see Appendix), were constructed to reflect plausible clinical profiles and trade-offs. Because the present study focused on clinician interaction with alternative weighting schemes rather than on developing or validating a new predictive model, the simulated probabilities were held constant across experimental conditions and were not evaluated as a standalone prediction method against published algorithms. The specific side effects were selected based on their high prevalence as drivers of non-adherence (11, 12, 28).

We examined three distinct weighting schemes for aggregating these predictions:

#### 2.3.1 Condition 1: implicit weighting (probabilities alone)

**Figure 2:**
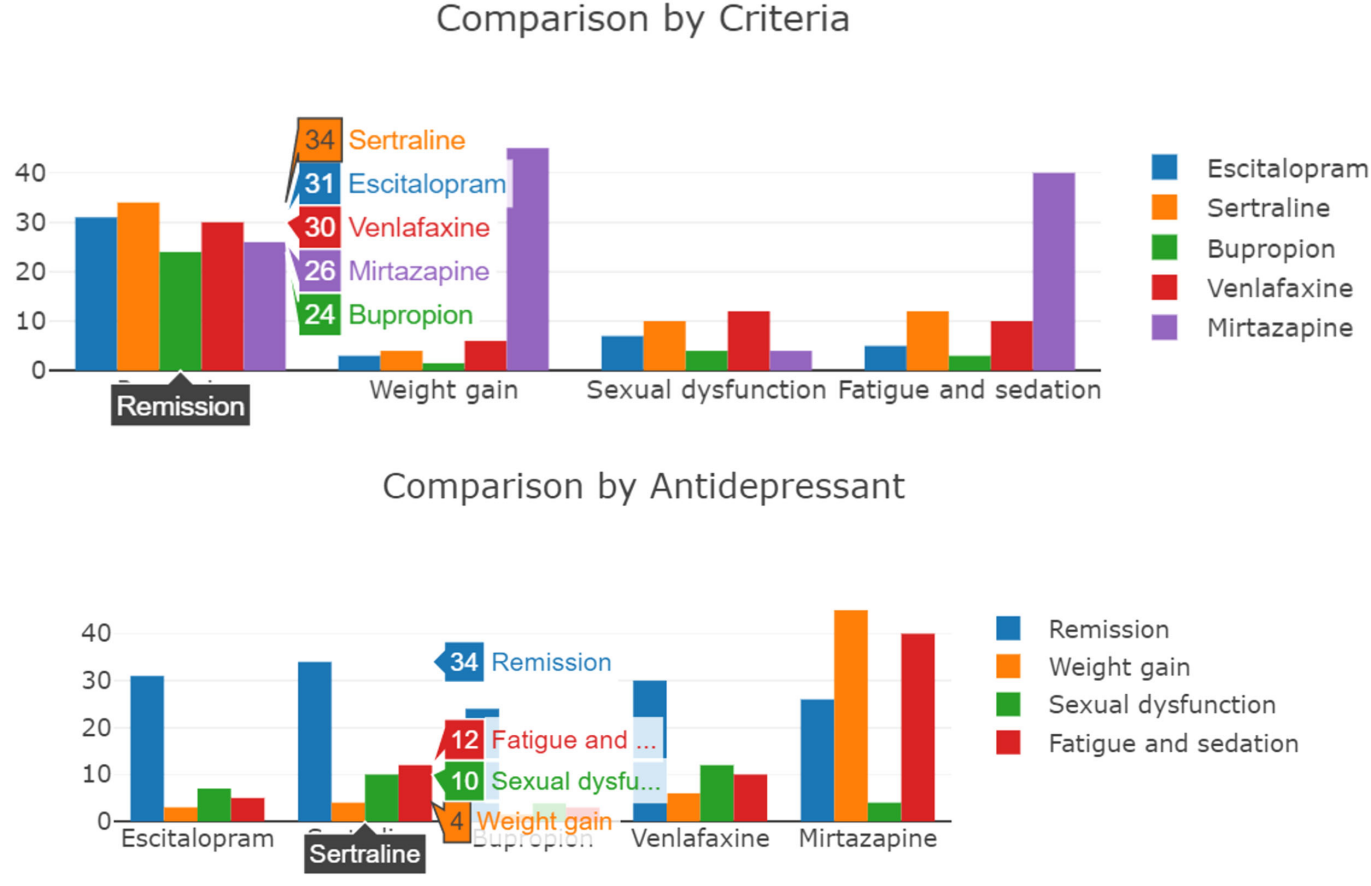
Implicit weighting. Interactive charts compare drugs by individual outcomes (e.g., remission probability) or by drug profiles. No aggregated score is provided.

In this setting, the system displayed raw probability estimates for remission and side effects via interactive 116 bar charts (**Figure 2**) and tabular data. No aggregated score was provided. This baseline mimics standard 117 “dashboard”-like analytics, forcing the clinician to perform the complex aggregation implicitly (cognitively) 118 without algorithmic assistance.

#### 2.3.2 Condition 2: static expert-derived weighting

**Figure 3:**
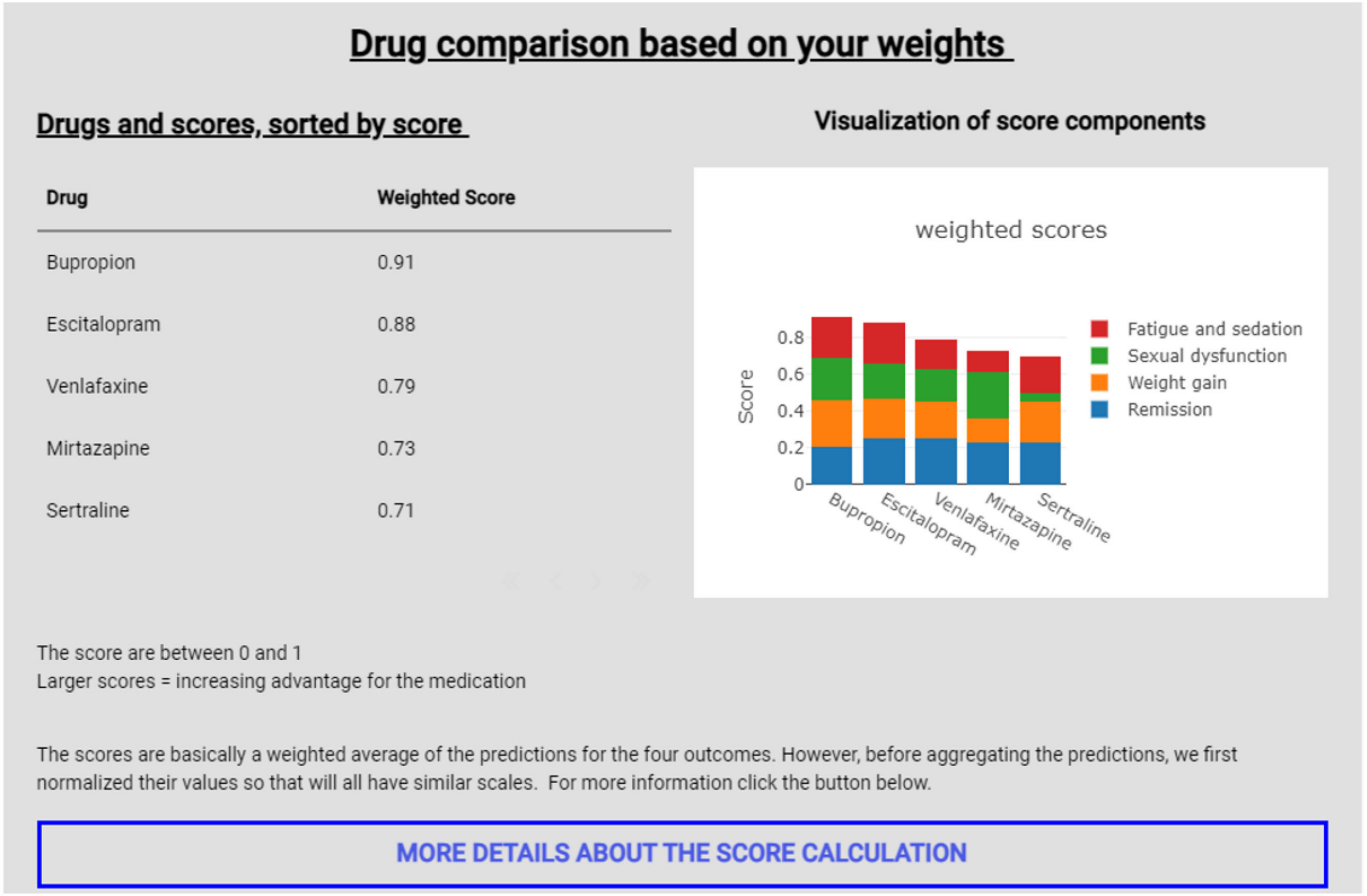
Expert-derived weighting. Drugs are ranked by their aggregated $S_{j}$scores. Stacked bars provide explainability by showing the contribution of each weighted criterion to the total score.

The Expert-Derived Weighting (EDW) scheme implements a weighted sum model to calculate an aggregated score *S_j_* for each drug *j*, calculated as follows:


$$S_{j}=\frac{\sum_{i=1}^{n}w_{i}\cdot v_{ij}}{\sum_{i=1}^{n}w_{i}}$$

where $v_{ij}$ is the normalized predicted value of outcome i for drug j, and $w_{i}$ represents the weight assigned to that outcome. The five drugs were presented as a ranked list sorted by $S_{j}$, with explainability supported via stacked bar charts decomposing the contribution of each criterion to the total score (**Figure 3**). Importantly, the weights *w_i_*were pre-defined by a panel of three senior psychiatrists who reviewed the patient profiles and deliberated to reach a consensus on the optimal weights for each case. The integration of static weights simulates the “black-box” nature of many AI-CDSSs, where value alignment is determined by external domain knowledge rather than the user.

#### 2.3.3 Condition 3: dynamic clinician-determined weighting

**Figure 4:**
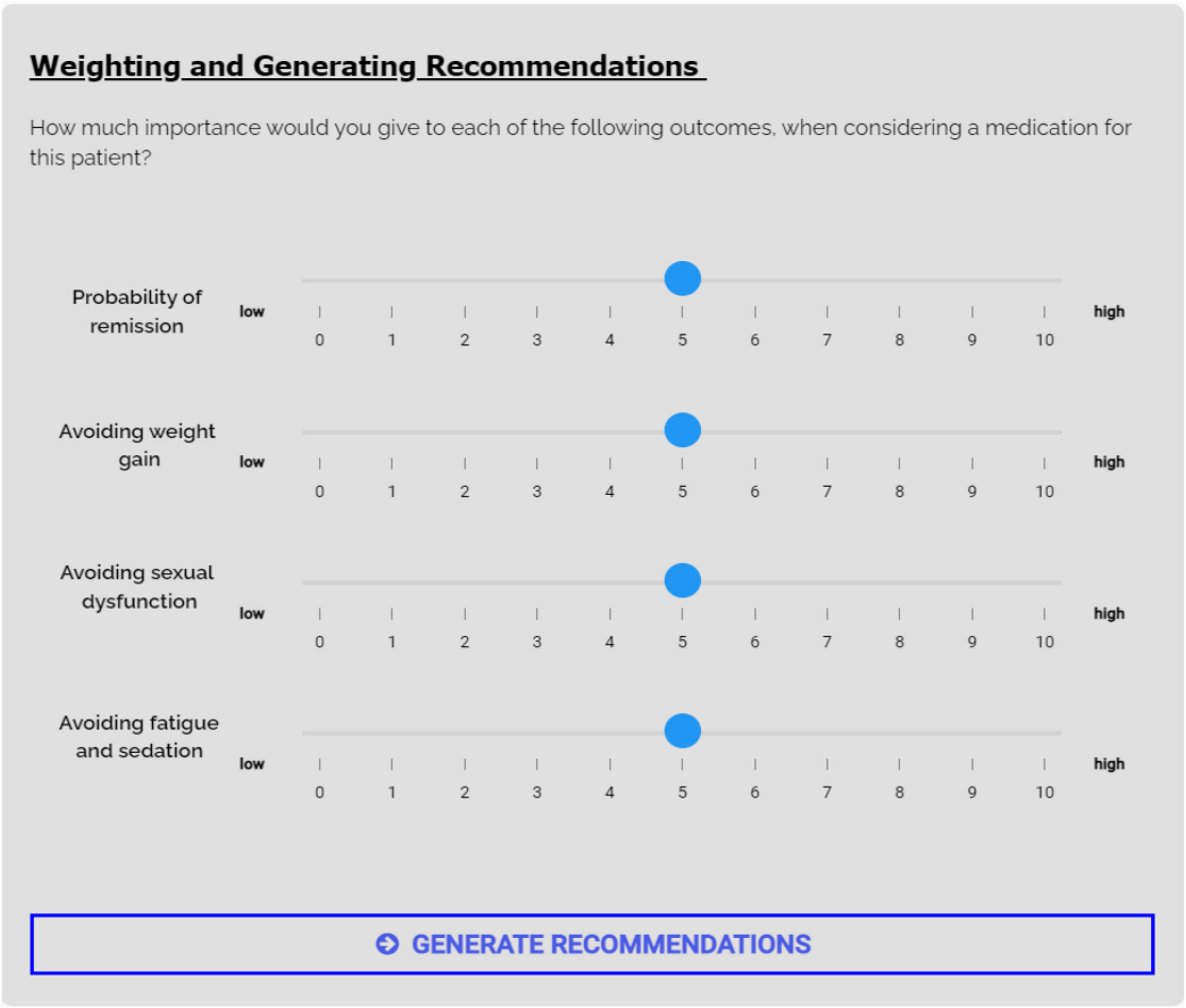
Clinician-determined weighting. Drugs are ranked by their $S_{j}$ scores, which are adjusted dynamically as the user modifies the importance weights via interactive sliders.

The Clinician-Determined Weighting (CDW) scheme follows the same technical and visualization approach as the EDW condition but shifts the locus of control to the user. Participants explicitly assigned weights to each criterion using interactive sliders (**Figure 4**). Following any adjustment, the system automatically recalculated the *S_j_*scores, updating the drug rankings and visualizations in real-time to reflect the clinician’s specific preferences.

### 2.4 Participants

We recruited 28 clinicians (17 psychiatrists and 11 primary care physicians/family doctors), primarily through professional networks in Israel, Canada, and the USA. Recruitment was conducted via email and LinkedIn. Of these, 22 participants (*N* = 22; 13 psychiatrists, 9 PCPs) completed the full protocol and are included in our analysis. Participants self-reported their home country as Israel (*n* = 10), the USA (*n* = 6), Canada (*n* = 4), and Other (*n* = 2). The two participants in the Other category were recruited through a US-based university setting but self-identified European countries as their home countries. The study was approved by the Committee for the Approval of Research Involving the Participation of Human Subjects at Bar-Ilan University.

### 2.5 Analysis

We primarily analyze quantitative outcome measures and supplemented these analyses with exploratory qualitative feedback from optional interview components.

#### 2.5.1 Quantitative analysis

We evaluated four primary metrics:

1. Perceived Clinical Utility: Assessed via post-phase questionnaire using a 5-point Likert scale.
2. Perceived Impact on Decision Making: Assessed via post-phase questionnaire using a binary item.
3. Time Spent: We measured the time the participants spent using each of the three schemes.
4. Observed Impact on Decision Making: Automatically measured as a binary variable of “treatment change” –this was defined by comparing the participant’s initial *unassisted* choice (recorded prior to interacting with the AI-CDSS) with their *final* choice (recorded after interacting with the weighting scheme).

Due to the non-normal distribution of the data and the sample size (*N* = 22), we utilized the non-parametric Kruskal–Wallis test (29) for main effects across the three conditions, followed by Dunn’s test (30) for *post-hoc* pairwise comparisons.

#### 2.5.2 Qualitative analysis

To deepen our understanding of user perceptions, participants were offered the opportunity to participate in a semi-structured interview following the experiment, debriefing them on their experience with the three weighting schemes. Three participants (*n* = 3) agreed to take part in this optional phase. Given the small number of interview participants, the qualitative analysis was treated as exploratory and intended to provide illustrative insights into user attitudes toward clinical utility and agency rather than definitive thematic conclusions. Themes were identified using inductive thematic analysis (31) and validated via investigator triangulation (32).

## 3 Results

In this section, we present both the quantitative and qualitative results.

### 3.1 Quantitative results

#### 3.1.1 Participant demographics

Our final sample consisted of 22 participants (13 psychiatrists, 9 PCPs) who completed the full protocol. **Table 2** details the demographic and professional characteristics of the cohort.

**Table 2:** Participant demographics (*N* = 22).

Attribute	Category	Count (Mean ± SD)
Age	–	47.3±8.53
Gender	Female	11
	Male	11
Experience (Years)	0–4	2
	5–9	6
	10–14	2
	15+	12
Specialty	Psychiatry	13
	Primary Care (PCP)	9
Home Country	Israel	10
	USA	6
	Canada	4
	Other	2

#### 3.1.2 Perceived clinical utility

Participants rated the clinical utility of each weighting scheme on a 5-point Likert scale (1=Very Little, 5=Very Much). A significant main effect of the weighting scheme on perceived utility was observed (*H*(2) = 10.29*, p <* 0.01). *Post-hoc* analysis indicated that the CDW scheme (Mean: 4.18 ± 0.89) was rated significantly higher than both the Implicit Weighting baseline (Mean: 3.27 ± 0.86*, p <* 0.01) and the EDW scheme (Mean: 3.41 ± 1.19*, p <* 0.05).

**Figure 5:**
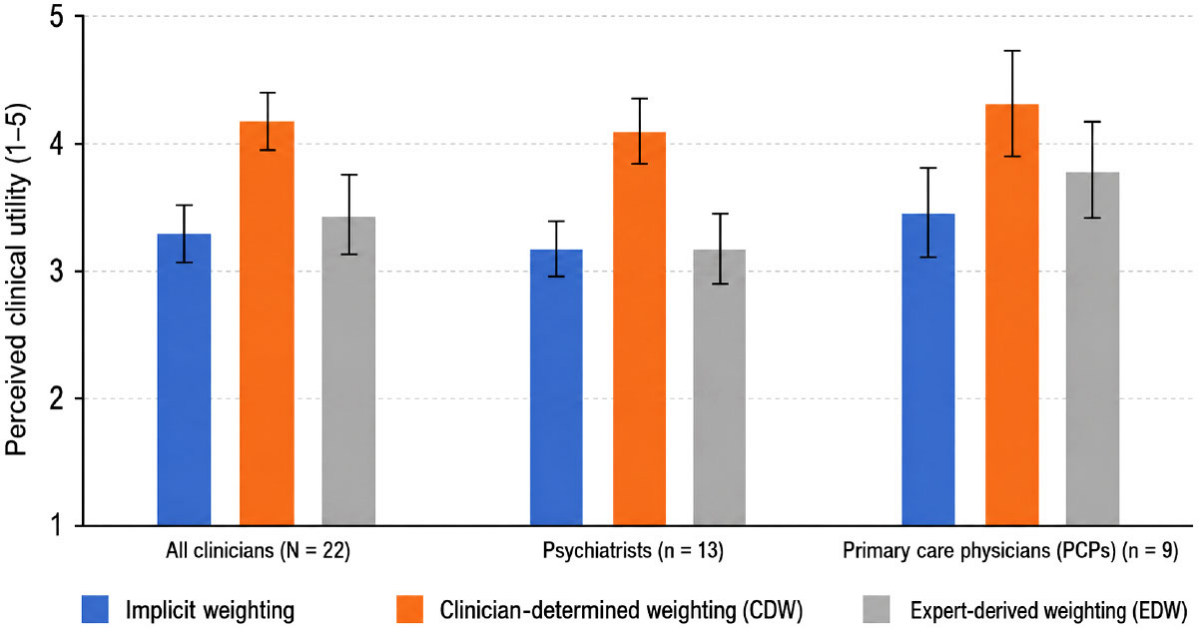
Perceived clinical utility. Mean perceived clinical utility ratings for the three weighting schemes across the full sample and by clinician specialty subgroup. Higher values indicate greater perceived clinical utility. Error bars indicate the standard error of the mean.

Subgroup analysis (**Figure 5**) showed that psychiatrists significantly preferred the CDW scheme over both alternatives (*H*(2) = 8.42*, p <* 0.05). PCPs also rated the CDW the highest (Mean: 4.33), though the difference did not reach statistical significance in this subgroup.

#### 3.1.3 Perceived impact on decision making

Participants self-reported whether the interaction with the AI-CDSS changed their initial decision in each phase (**Table 3**). While 54.5% reported an affirmative impact in the Implicit Weighting baseline, this was driven largely by PCPs (77.8%) rather than psychiatrists (38.5%). In contrast, the CDW scheme showed a consistent impact across both groups (53.8% of psychiatrists, 66.7% of PCPs). The EDW scheme had the lowest reported impact (36.4% overall), particularly among psychiatrists (23.1%).

**Table 3:** Perceived impact on decision.

Weighting scheme	All	Psychiatrists	PCPs
Implicit (Baseline)	54.5%	38.5%	77.8%
EDW	36.4%	23.1%	55.6%
CDW	**59.1%**	**53.8%**	66.7%

Percentage of participants indicating that the weighting scheme influenced their treatment choice (affirmative response).

#### 3.1.4 Time spent

While no statistically significant time differences were observed, the highest mean time was spent using the Implicit weighting scheme, with a mean of 404.7 sec (std of 343.8). For comparison, the mean time for using the CDW scheme was 355.8 sec (std of 162.75) and for the EDW scheme 320.6 sec (std of 154.1).

#### 3.1.5 Observed change in treatment

Across all 66 trials (*N* = 22 participants × 3 phases), an initial choice was recorded in 84.8% of cases. Of these, the CDW scheme prompted the highest rate of treatment revision: participants changed their initial choice in 33.3% of cases. In comparison, the *Implicit* baseline prompted a change in 26.3% of cases, and the EDW scheme in only 15.8% of cases.

### 3.2 Qualitative results

Three themes were identified from the interview transcripts: (1) Clinical Utility & Agency, (2) Integration Within Workflow, and (3) Transparency vs. Automation. Given the small qualitative subsample (*n* = 3), the following findings should be interpreted as exploratory and illustrative.

#### 3.2.1 Clinical utility & agency

Participants generally favored the CDW for its ability to operationalize their specific clinical intent. As one participant noted: “It made me consider the overall fit of the medication … rather than focusing on one aspect that might not have fit very well”. Conversely, the EDW faced resistance, particularly from psychiatrists. One remarked that weighting outcomes is “precisely the clinical work that psychiatrists should be doing,” viewing the static weights as an encroachment on their expertise. However, a minority preference for EDW was noted; one PCP preferred the EDW because “it felt like magic” and questioned the point of AI if physicians had to set weights themselves.

#### 3.2.2 Integration within the workflow

The CDW scheme was described as a means for *confirmation* and *refinement*. One clinician explained: “It confirmed the decision I had in mind … made me more confident I was not overlooking important information”. Another participant stated, “I changed the initial treatment based on the side effects” after seeing the weighted impact.

#### 3.2.2 Transparency vs. automation

Resistance to the “black box” nature of the EDW scheme was observed. Participants expressed that expert-set weights “are no substitute for engaging patients in their own care,” highlighting that the EDW scheme contradicts shared decision-making principles. In contrast, the CDW scheme was seen as a potential bridge for patient communication: “Patients would love to see how their doctor comes to their decision”.

## 4 Discussion

Our results demonstrate a comparatively consistent hierarchy of clinical utility where both specialists and PCPs preferred the CDW scheme over both the Implicit and EDW alternatives. The preference for the clinician-determined scheme suggests that while physicians value transparency, they also require tools that help synthesize complex clinical profiles. Standard “dashboard” analytics, while common, burdens clinicians with the cognitive load of mentally integrating competing side-effect probabilities. The dynamic scheme offloads this burden while protecting the physician’s therapeutic intent and agency.

Crucially, the preference for the CDW scheme over the EDW scheme highlights the limitations of “black-box” value alignment, agreeing with a long line of works in the field of human-computer interactions (33, 34). Two key mechanisms likely drive this result. First, clinicians, particularly specialists, resisted the static model because it encroached on their core expertise—determining the “value” of a clinical outcome for a specific patient, aligning with (35, 36). By restoring the locus of control to the user, the CDW scheme transformed the AI from a “replacement” to an “assistant”, thereby increasing acceptance. Second, in our exploratory qualitative feedback, the CDW scheme was described as serving as a potential boundary object between clinician and patient, allowing for collaborative weighting of side effects - a workflow impossible with rigid expert weights (17, 37).

Our findings regarding decision impact, both perceived and observed, further underscore this dynamic. The CDW scheme was not only the most preferred but also the most effective at prompting an AI-informed treatment decision (33.3% change rate). Interestingly, the EDW scheme had the *lowest* impact on decision-making (15.8%), particularly among psychiatrists. This suggests that when AI seemingly “dictates the rules” of the decision (via fixed weights), experts are more likely to dismiss the recommendation entirely and maintain their agency. Conversely, when experts define the rules (via the CDW scheme), they are more willing to accept the AI’s predictions (even if these are “black-box”), even when it does not align with their initial intuition. The time-spent results also provide preliminary evidence regarding real-time usability. Although the CDW scheme required active clinician input, it did not substantially increase interaction time compared with the Implicit or EDW schemes, suggesting that clinician-controlled weighting may be feasible within a short decision-support interaction.

Finally, the preliminary trend indicating a discrepancy between PCPs and psychiatrists hints at a potential “Floor-Ceiling” effect in AI-CDSS (38, 39). While larger cohorts are needed to confirm this divergence, our initial data suggest that PCPs, who may have less domain expertise in psychopharmacology, derived high value from both the implicit and CDW schemes. Psychiatrists, conversely, found little value in the implicit weighting scheme but rated the CDW scheme highly. This implies that flexible, controllable AI architectures are essential for engaging domain experts who might otherwise reject decision support (40).

It is important to note that our study has several limitations that offer potentially fruitful avenues for future work. First, the sample size (*N* = 22) limits the statistical power for subgroup analyses, particularly between specialties. While non-parametric tests confirmed main effects, larger cohorts could bring about more nuanced results, such as validating the PCP-Psychiatrist divergence. In addition, the qualitative interview component included only three participants and should therefore be interpreted as exploratory and hypothesis-generating rather than as evidence of thematic saturation. Second, the study utilized simulated vignettes rather than real patient interactions. While this controlled for case variance, it cannot fully capture the pressures of a live clinical environment or the direct input of a patient in the loop. Third, our implementation of the EDW scheme relied on a single set of three experts. It is thus plausible that a more comprehensive expert evaluation could bring about more acceptable expert-based weights. Fourth, although user interaction time was measured as an exploratory proxy for real-time usability, the prototype used pre-defined probability estimates and did not evaluate computational performance metrics such as model inference time, system latency, throughput, or integration with real-time electronic health record workflows. Finally, both the CDW and EDW schemes assume linear independence between criteria, which is clearly a simplification of reality, and the CDW scheme additionally requires clinicians to explicitly express patient-specific priorities as numerical weights.

It is important to note that the proposed CDW approach has several limitations. The method assumes that clinicians can explicitly express patient-specific priorities as numerical weights, which may oversimplify the complexity of shared decision-making. The weighted-sum model also assumes linear and independent trade-offs between remission probability and side-effect risks, whereas real clinical preferences may involve threshold effects, interactions among outcomes, or non-linear risk tolerance.

## 5 Conclusion

This exploratory pilot study examined how different weighting schemes in an AI-CDSS influence physicians’ perceived utility and antidepressant treatment decisions. Compared with implicit presentation of probabilities and static expert-derived weighting, the dynamic clinician-determined weighting scheme was rated as the most clinically useful and produced the highest rate of observed treatment revision. These findings suggest that clinician-facing AI-CDSS tools may be more acceptable and impactful when they allow physicians to explicitly control how predicted benefits and side-effect risks are weighted for a given patient.

The results should be interpreted cautiously given the modest sample size, simulated vignette design, and exploratory qualitative component. Nevertheless, the study highlights an important design consideration for AI-augmented psychiatric decision support: predictive accuracy alone is insufficient if clinicians cannot align recommendations with patient-specific priorities and clinical judgment. Future work should evaluate clinician-controlled weighting mechanisms in larger samples, real clinical workflows, and systems using live predictive models.

## Data Availability

The raw data supporting the conclusions of this article will be made available by the authors, without undue reservation.

## Appendix: clinical scenarios

**Case 1: Jack (Mild-Moderate Depression)**

Profile: 82-year-old male, retired, lives alone (widowed/separated). Generally active social life (senior’s club) but withdrawing recently. Medical History: Hypertension and Type II Diabetes (well-controlled). No prior psychiatric history. Presenting Symptoms:

- Mood: “Feeling sad”, “not myself”, loss of enjoyment in hobbies (cards).
- Anxiety: Recent panic attacks during decision-making; observed fidgeting/tremors.
- Vegetative: Difficulty falling asleep, early morning awakening, increased fatigue.
- Physical: Dizziness upon standing (orthostasis), occasional skin itchiness.

**Preferences/Concerns:**

- Low concern for sexual side effects (“not active anymore”).
- High concern for weight gain (diabetes risk).
- Generally adherent to medications (takes BP pills without issue).

**Diagnosis:** Mild-Moderate Depression. No suicidality.

**Case 2: Emma (Moderate Depression)**

Profile: 40-year-old female, lawyer/consultant, married. High-functioning professional facing recent work stress. Medical History: None. Presenting Symptoms:

- Mood: Irritable, sad, feeling guilty (“letting people down”), sobbing during interview.
- Cognitive: Difficulty concentrating on reading/work.
- Suicidality: Passive ideation (“better if I were dead”) but no intent/plan.
- Vegetative: Middle insomnia, weight loss (2 lbs), fatigue.
- Somatic: Health anxiety (fear of cancer), constipation.

**Preferences/Concerns:**

- High concern for sexual dysfunction (marital strain).
- High concern for weight gain.
- Anxious about side effects in general (medication na¨ıve).

Diagnosis: Moderate-Severe Depression.

**Case 3: Sara (Severe Depression)**

Profile: 51-year-old female, unemployed (lost job in food service due to symptoms), lives alone. Medical History: None known. Presenting Symptoms:

- Mood: Depressed mood over 50% of time, significant anhedonia (unable to work).
- Psychomotor: Significant slowing (latency in speech).
- Cognitive: Severe indecisiveness, inability to manage finances.
- Suicidality: Frequent suicidal ideation (several times a week), wishes she were dead, but no active plan.
- Vegetative: Severe insomnia (initial, middle, and late), weight loss (5 lbs).

**Preferences/Concerns:**

- Difficulty articulating specific concerns due to severity of illness.
- General worry about side effects; requires prompting.

**Diagnosis:** Severe Depression.
